# Adaptations of an Online Cognitive-Behavioral Therapy Intervention for Binge-Purge Type Eating Disorders in Publicly-Insured and Uninsured Adults: A Pilot Study

**DOI:** 10.21203/rs.3.rs-3879484/v1

**Published:** 2024-02-02

**Authors:** Siena S. Vendlinski, Agatha A. Laboe, Peyton Crest, Claire G. McGinnis, Molly Fennig, Denise E. Wilfley, C. Barr Taylor, Ellen E. Fitzsimmons-Craft, Erin C. Accurso

## Abstract

Background Publicly-insured and uninsured individuals—many of whom are marginalized because of race/ethnicity, disability and/or sexual preferences—experience barriers to accessing evidence-based interventions for eating disorders (EDs). Additionally, EBIs have not been developed with or for diverse populations, exacerbating poor treatment uptake. Mobile technology is perfectly positioned to bridge this gap and increase access to low-cost, culturally-sensitive EBIs. Methods This study leverages a user-centered design approach to adapt an existing coached cognitive-behavioral therapy-based digital program and evaluate its usability in a sample of 11 participants with (sub)clinical binge-purge type EDs who are publicly-insured ( *n* = 10) or uninsured ( *n* = 1). Participants were primarily Non-Hispanic White ( *n* = 8) women ( *n* = 8). Two semi-structured interviews occurred with participants: one to assess treatment needs and the other to obtain app-specific feedback. Interviews were coded using inductive thematic analysis. Results Interview 1 feedback converged on three themes: Recovery Journey, Treatment Experiences, and Engagement with and Expectations for Online Programs. Participants endorsed facing barriers to healthcare, such as poor insurance coverage and a lack of trained providers, and interest in a coach to increase treatment accountability. Interview 2 feedback converged on three themes: Content Development, Participant Experiences with Mental Health, and Real-World Use. Participants liked the content but emphasized the need to improve diverse representation (e.g., gender, body size). Conclusions Overall, user feedback is critical to informing adaptations to the original EBI so that the intervention can be appropriately tailored to the needs of this underserved population, which ultimately has high potential to address critical barriers to ED treatment. Trial Registration This study was reviewed and approved by the Institutional Review Board (IRB) at the University California, San Francisco (IRB #22-35936) and the IRB at Washington University in St. Louis (IRB ID 202304167).

### Background

Eating disorders (EDs) are severe psychiatric illnesses characterized by disrupted eating behaviors and excessive preoccupation with weight, food, and body image that interfere with functioning. EDs affect individuals across race, ethnicity, and socioeconomic status (SES), and do not typically differ by race or ethnicity, except for bulimia nervosa which is more prevalent among Latino and Black vs White individuals (1–3). Unfortunately, < 20% of individuals with EDs ever receive care, with treatment uptake being lowest amongst those who do not identify as female, White, non-Hispanic, or high income (4, 5).

Low treatment uptake is exacerbated by poor treatment access, particularly for individuals from under-resourced communities, including those with low SES, racial and ethnic minorities, and those with Medicaid insurance (5–9). Numerous barriers to care are salient, such as the high costs of participating in specialized mental health services (10, 11). Individuals with public insurance are often unable to access high-quality treatment because providers in publicly-funded settings are not trained in treating EDs (12). Furthermore, screening for EDs in public mental health systems is poor (13). As such, innovative solutions, such as digital interventions, are needed to increase detection and treatment of EDs among individuals who are high risk for health inequities (14).

Digital interventions may be especially beneficial for individuals from lower SES backgrounds given that they reduce logistic and financial barriers to care (e.g., travel, work schedule, flexibility with timing of use). Publicly-insured adults have relatively high digital access, with 86% owning a smartphone and 79% having access to broadband internet in their household (15, 16). However, standard ED treatments have not yet been adequately tailored to meet the unique needs of individuals from lower SES and racially or ethnically diverse backgrounds. Thus, cultural adaptation is critical and has been found to improve treatment relevance, acceptability, effectiveness, and sustainability of evidence-based interventions (EBIs) (12, 17–19).

Cultural adaptations may be particularly impactful when informed by user-designed approaches (otherwise known as human-centered design or design thinking). User-centered design focuses the design of technology on individuals who will be using it and the contexts in which it will be implemented. It is particularly effective when conducted in iterative phases and generally includes usability testing, which assesses whether the product functions as intended (20). In line with a user-centered design approach, this study developed and evaluated the usability of a guided self-help, cognitive-behavioral therapy (CBT)-based digital program—a guideline-based, first-line treatment for binge-purge type EDs in adults –for use among individuals with EDs who are publicly insured or uninsured (21).

### Methods

## Participants and Recruitment

Eligibility criteria required that participants were at least 18 years old, screened positive for a clinical or subclinical binge-purge type ED, had a BMI ≥ 18.5, and were publicly-insured (e.g., Medicaid, Medi-Cal, or Medicare) or uninsured. Participants were also required to speak and read English, reside in the United States, and have access to an electronic device. Participants were primarily recruited through the online ED screening tool hosted by National Eating Disorders Association. Additional recruitment was facilitated through social media, community health partners, and ED advocacy groups.

## Procedure

Verbal consent was obtained from eligible participants prior to enrollment. Participants completed two one-hour, semi-structured virtual interviews—a needs assessment interview and a usability testing interview (see Supplemental Information for interview guides)—which focused on potential adaptations to adequately address context and related challenges specific to individuals with EDs with fewer resources (e.g., finances, time, education) and diverse social identities (e.g., gender, race/ethnicity, ability status, and weight status).

## Needs Assessment

Needs assessment questions gathered data on past ED treatment experiences (or lack thereof), current goals related to ED recovery, the role of technology in everyday life, and how technology can be harnessed to support mental health generally and ED recovery specifically.

## Usability Testing

Participants who completed the needs assessment interview completed usability testing one to three months later, depending on the phase of the study. One usability testing interview was done in a group of two participants; all others were completed one-on-one with the interviewer. Participants were shown screenshots of different modules and provided feedback on content, intervention design, aesthetics/layout, clarity, accessibility, and perceived usefulness of the intervention.

## Online Intervention Design

The web-based intervention, *Changing Attitudes, baLance, and Mindfulness for Eating Disorders (CALM-ED)*, was adapted based on an existing CBT-based online program shown to be effective in reducing ED behaviors in a population of college women with binge-type EDs (22–24). CALM-ED is comprised of eight CBT-informed modules: Eating Well, Coping Well, Thinking Well, Mind and Body Wellness, Media Wellness, Relationship Wellness, Emotional Wellness, Future Wellness. Each module includes educational content with infographics, interactive activities, and supplemental materials (e.g., downloadable PDFs). See Supplemental Information for an overview of each module. Hosted on a web-based platform, CALM-ED can be accessed from both mobile devices and computers (refer to Fig. 1 and Fig. 2 for screenshots).

## Qualitative Thematic Analysis

The study team transcribed interview recordings and then used qualitative inductive analysis to identify repeating patterns within the contexts of participant feedback. In line with best practices, coders first discussed their positionality. Then, following steps identified by Braun & Clark[24], the study team analyzed transcripts through a multi-step process: the study team read the transcripts, created separate codebooks for the needs assessment and usability testing, coded the transcripts (two independent coders on each transcript), and identified, defined and named the themes (25).

### Results

## Participants

We expected that the 11 participants enrolled sufficed for the purposes of qualitative analyses since sample sizes above nine often achieve coding saturation (26, 27). Adult participants were primarily cisgender females (*n* = 8, 72%) who identified as White and Non-Latinx (*n* = 8, 72%) with a household income of less than $40,120 (*n* = 9, 81.8%). See Table 1 for study participant details.

Table 1
Participant Demographics.

| Participant # | Age (years) | Race and Ethnicity | Gender Identity | Diagnosis | Sexual Orientation | Household Income (thousands) | Insurance Status | At Risk for Food Insecurity |
|---|---|---|---|---|---|---|---|---|
| 1 | 21 | White, Non-Latinx | Female | Clinical/Subclinical Atypical Anorexia, Binge/Purge | LGBQ+ | Less than $12,880 | Public | Yes |
| 4 | 52 | White, Non-Latinx | Female | Clinical/Subclinical Atypical Anorexia, Binge/Purge | LGBQ+ | $21,961-$26,500 | Public | Yes |
| 5 | 34 | White, Non-Latinx | Female | Clinical/Subclinical Atypical Anorexia, Binge/Purge | Heterosexual | $21,961-$26,500 | Public | No |
| 6 | 36 | White, Non-Latinx | Female | Clinical/Subclinical Atypical Anorexia, Binge/Purge | Heterosexual | $26,501-$31,040 | Public | Yes |
| 7 | 22 | Black or African American, Non-Latinx | Non-binary | Clinical/Subclinical Atypical Anorexia, Binge/Purge | LGBQ+ | $12,881-$17,420 | Public | Yes |
| 8 | 29 | White, Non-Latinx | Male | Clinical/Subclinical Atypical Anorexia, Binge/Purge | LGBQ+ | Less than $12,880 | Public | Yes |
| 9 | 29 | Black or African American, Non-Latinx | Female/Non-binary | Subclinical Bulimia Nervosa | LGBQ+ | $35,581-$40,120 | Public | Yes |
| 11 | 24 | White, Non-Latinx | Female | Subclinical Bulimia Nervosa | Heterosexual | $35,581-$40,120 | Public | Yes |
| 12 | 70 | White, Non-Latinx | Female | Unspecified feeding or eating disorder | Heterosexual | $65,001-$100,000 | Public | No |
| 13 | 68 | White, Non-Latinx | Female | Binge Eating Disorder | Heterosexual | $65,001-$100,000 | Public | No |
| 14 | 38 | American Indian or Alaska Native, Latinx | Female | Subclinical Bulimia Nervosa | Heterosexual | Less than $12,880 | Uninsured | Yes |

## Needs Assessment

Feedback converged on three main themes. See additional examples of feedback displayed in Supplementary Table 2.

## Recovery Journey

**Consequences of the ED**. Participants exhibited self-awareness of their ED, highlighting consequences of their EDs across multiple areas of their lives, including social, physical, occupational, and emotional domains. One participant shared, “I didn’t have any friendships. I realized I didn’t have any skills to live on my own—how to pay bills, how to deal when things went wrong—just daily tasks I couldn’t cope with those without wanting to fall back on behaviors. I didn’t have any plans for my future,” (P8) voicing the serious toll his ED took on everyday functioning. Another participant explained, “[My ED] has impacted my life. It’s impacted my relationships and how I view myself, and probably my professional life as well” (P6).

**Goals for Recovery.** Participants reported high motivation to pursue recovery in order to feel better about themselves and improve their quality of life. Goals often included remediating the consequences of their EDs, stopping ED behaviors, and developing new coping mechanisms. One participant noted, "I'd like to completely stop the binge-purge cycle and just be able to eat more throughout the day" (P5). Another participant commented on the importance of being able to use skills effectively: "I feel like [developing] skills would probably be my most important goal right now...like these are the skills that I can use when I have the urge to restrict" (P1).

## Treatment Experiences

**Barriers to Treatment Initiation and Uptake.** Participants reported numerous barriers to ED treatment uptake, including lack of insurance coverage, lack of geographic accessibility, and discrimination, which often intersected. One participant shared, "[Higher levels of care are] just not attainable—like, we have looked at every option—because of the type of insurance [Medicaid/Medicare] I have." (P1). Another participant expressed, "As far as...finding...an outpatient or an inpatient program, that was difficult.... A lot of places—because I was overweight or my BMI was higher than a certain amount, even though I met other criteria—were like, 'I don't think this is the right fit'" (P4).

**Poor Quality ED treatment.** Even when participants were able to receive ED treatment, many lamented poor quality of treatment, largely due to a lack of trained providers. One participant shared, "My current therapist is...not specifically an eating disorder therapist...[Good eating disorder therapists] are few and far between" (P7). Similarly, another participant noted, "With the therapist I see now..., I just feel like she doesn't get it....There's definitely a difference between someone with the special licenses [to treat EDs] and someone who doesn't have it" (P4).

**General Treatment Experiences.** Finally, participants shared experiences with treatment for other ED-related physical and mental health diagnoses. Participants discussed the difficulty of managing physical illnesses, such as diabetes and chronic liver injury, in the context of an ED. One participant underwent bariatric surgery, describing subsequently elevated ED cognitions in the aftermath that ultimately led to an ED relapse; others were contemplating bariatric surgery in the future. Additionally, participants reported co-occurring anxiety and/or depression, which were often the focus of treatment and precluded the ED from being addressed.

## Experiences with and Expectations for Online Programs

**Past and Present Experiences with Technology.** Participants endorsed using multiple health-related apps, including My Fitness Pal, My Strength, Recovery Record, Weight Watchers, and Finch, as well as health technology, such as Fitbits and Apple watches. Participants had mixed feelings about whether technology had supported or impeded their ED recovery. For example, a participant shared, "I used to use My Fitness Pal. Not when I was like healthy dieting, or when it spiraled, but when I was trying to get...back on track for eating. I was trying to be a little more mindful of just...tracking it, but then that kind of backfired because when I saw it written out of like, 'Oh, you ate this!' Then it kinda just became detrimental instead of helpful" (P5), highlighting potential harms from app use despite recovery-oriented intentions.

Participants also shared aspects of the technology they did and did not like. One participant shared of Recovery Record (i.e., an app used to track meals alongside therapeutic support), "I think if I could take the time to do all of that, then it's helpful. But sometimes...it just takes too long to answer all the little questions" (P4), expressing dislike for the number of prompts. On the other hand, the same participant noted that "It's definitely better if you can connect it with someone who can look at it. Sometimes I think it's helpful just where they [treatment team] can just see it, but you don't have to like actually talk about, you know, as it's hard to admit" (P4).

**Future Wants and Needs for Online Program.** Participants also gave feedback on what they hoped to see in an online program designed to facilitate ED recovery. For example, one participant shared, "Reminders or messages to kind of keep it in focus [would help keep me on track]" (P9). Participants also frequently endorsed the value of accountability with a coach in the program or peers going through a similar struggle. Furthermore, participants provided content recommendations, including adding content around managing co-occurring mental health conditions that could trigger ED behaviors.

## Usability Testing

Usability testing feedback converged on three main themes. See additional examples of feedback displayed in Supplementary Table 3.

## Content Development

**Specificity.** In general, participants were enthusiastic about the range of topics covered, emphasizing that much of the content resonated with their experiences and was consistent with what they hoped to see in an online program. One participant shared, "I really like the goals, the values. I really like how the questions [about your relationships] made you really think about...who are these people and how you want them in your life, and you know, how you associate with them" (P14). Another participant explained, "I like the 'balanced self talk.' I like the examples of the 'critical self' because I think we don't really think about those things on the daily" (P13). Others highlighted the need to strengthen the connection between the skills presented and specific eating disorder-related challenges, stating, "…what I'm reading here, although it's great information, I'm not reading anything about this and an eating disorder…what can I do to help myself with my eating disorder?" (P13).

**Inclusivity.** Participants also commented on how to make the content more inclusive to people of various social identities. Many participants focused on the importance of representing people in bigger bodies and not just “the stereotypical eating disorder—like super skinny girl that thinks she’s fat, but she’s not” (P5). Participants also mentioned the importance of making the program relevant to men and gender diverse individuals.

**Usability.** Finally, participants offered suggestions to increase the usability of the program, including changing phrasing, breaking up text into smaller chunks, reducing the number of questions, and having audio options for long text passages. Participants also gave feedback about specific program tools. Regarding the meal tracking tool, one participant noted, “My first thought is...having a setting to click what are supposed to be your meal goals, because right now for me personally, we haven't got into snacks, and we're working on it” (P1), offering a suggestion to customize the tool. Overall, participants felt that the content was personally relevant and addressed an array of topics important for recovery; however, it did not necessarily reflect their social identities nor had the specificity needed to make the program feel tailored to eating and body related concerns.

## Participant Experiences with Mental Health

**ED Behaviors.** Participants’ own ED behaviors directly informed their suggestions for improvement and their overall feelings towards the program content. One participant suggested that “[The program should cover] options for destroying of a scale... just to reiterate the point that weighing yourself is not a good idea” (P5). Another participant liked how the program approached exercise: “So I really like…that this takes a really balanced view with exercise. I like that it gives a variety of options for exercise [and]…. it allows me to think about physical activity in any sort of way. Not just thinking about it in a traditional way of going to the gym, or whatever” (P8).

**Treatment Exposure.** Participants also shared their familiarity with different psychological skills and treatment modalities. For example, one participant expressed previous exposure to CBT and noted, “I feel that [identifying thinking patterns (e.g., catastrophizing)] is really useful.... I was in therapy before, for a couple of years and we talked about these kind of mentalities, and they’re definitely things that I had to deal with… to undo” (P14). Another participant indicated familiarity with the concept of meal tracking, and some concern about whether meal tracking would be focused on nutrition details and calories, or on feelings around eating (P7). Other elements of the program resonated as novel, as demonstrated by P4’s remarks on addressing physical activity: “I think I have not used another app, or anything that actually addresses exercise…in this way that I’m aware of. It might say, did you exercise or what did you do, but there’s nothing like, ‘Let’s come up with a plan.’” In general, participants found information presented in the program important and relevant, whether familiar or not.

## Real-World Use

**Previous App Use.** One participant noted a dearth of apps focused on alleviating ED symptoms, sharing, “There are a lot of mental health apps out there, but there are not a lot of apps that are specific for eating disorders and challenging negative body image but also other aspects of your life” (P1), underscoring the importance of an online program focused on addressing EDs and broader life challenges simultaneously. Another participant expressed hesitation to use the program based on previous experiences with Recovery Record, sharing, “I hated Recovery Record because there were so many questions” (P5).

**Anticipated CALM-ED Engagement.** Overall, participants indicated enthusiasm about using the program in their daily lives, and all endorsed that they would at least trial the program. One participant shared, “I totally would [use this program]. I think it’s different from… what I’ve used in the past.... I really like having a plan, whether it’s for like meal, planning, or exercise, or whatever” (P4). Another participant explained *how* she would use the program, noting, “Yeah, I definitely would use [the audio component of the program] because I spend a lot of time in my car, so if it was something that when I’m in my car, I usually listen to like podcasts and things like that” (P11).

### Discussion

The development and usability testing of a guided self-help digital CBT-based intervention for underserved adults with binge-purge type EDs fills a critical gap in advancing services for more diverse populations. The user-centered approach involved participants in each phase of the development process to increase relevance and prospective engagement, yielding several important insights (27). Many participants identified multiple difficulties accessing evidence-based care. Participants described feeling discouraged about the lack of trained ED providers and frustrated with having to explain their illness and educate their providers about EDs, aligning with past literature that has elucidated barriers to treatment faced by publicly and uninsured populations (9, 12). Notably, about half of the participants held one or more marginalized identities —most commonly queer, living with a physical disability, and having a larger body—which has been shown to pose unique challenges to receiving appropriate care (14).Geographical location further impacted participants’ ability to connect with treatment. Ultimately, there were many factors that contributed to negative experiences seeking and engaging in treatment, all of which bolstered participants’ desire for an evidence-based, ED specific digital intervention.

Participants' diverse identities and life circumstances affirmed that this program must address a breadth of unique obstacles faced by this population. In alignment with these experiences, feedback naturally centered around maximizing diversity and inclusivity throughout the program. Specifically, participants stressed the importance of including people with larger bodies and different abilities in examples of skills and accompanying infographics. They also emphasized improving accessibility throughout the program design, encouraging the addition of audio recordings and using graphics to streamline text-heavy sections, which could be reflective of education level (27). Participants were excited about the coaching component, which has previously been demonstrated to facilitate program engagement, [29] and wanted to connect with other users who have endured similar challenges to alleviate feelings of isolation (22). A desire to cultivate a sense of community within the program, even if users weren't directly engaging with others (e.g., direct messaging), reinforces the importance of social support in promoting recovery.

Limitations of this study include difficulties reaching the target population due to the complexities of public health care systems and potential sample bias. Given the known low rates of treatment seeking and engagement within the target population, this sample may have had unusually high treatment engagement and additionally did not reflect racial and ethnic diversity (5, 7, 29). Lastly, the user-centered design approach could not be responsive to initial feedback on aesthetics and delivery (e.g., customizable color schemes, a phone app delivery) due to financial limitations and use of the Qualtrics platform.

### Conclusions

To date, this is the first digital CBT-based intervention adapted for populations at risk of experiencing health inequities, using an original program that has already been shown to be effective in other populations. A user-centered design approach was used, which enhances probable future engagement, and well-established guidelines for qualitative analyses were utilized (25, 28). Finally, the participant pool included diversity in terms of age, geographic location, and income, capturing the experiences and perspectives of those who are under-resourced and underrepresented.

Preliminary results from the present study mirror emerging literature that mobile technologies have the ability to increase accessibility of treatment for those who have been historically precluded from accessing evidence-based ED care (28). Participant feedback highlighted several key areas of focus in order to adapt existing EBIs to be appropriate for and acceptable to underserved diverse populations. These findings inform future program development and refinement, with the potential to address critical barriers to ED treatment for a population that is most in need of tailored evidence-based care.

### Abbreviations

**ED(s)**
Eating disorder(s)
**CBT**
Cognitive-Behavioral Therapy
**EBI(s)**
Evidence-based intervention(s)
**SES**
Socioeconomic status
**BMI**
Body mass index
**IRB**
Institutional Review Board
**CALM-ED**
Changing Attitudes, baLance, and Mindfulness for Eating Disorders

### Declarations

#### Ethics approval and consent to participate

This study was reviewed and approved by the Institutional Review Board (IRB) at the University California, San Francisco (IRB #22-35936) and the IRB at Washington University in St. Louis (IRB ID 202304167). All participants engaged in an informed consent discussion prior to participating in any study procedures and freely provided their verbal informed consent.

#### Consent for publication

Not applicable.

#### Availability of data and materials

The full datasets generated and analyzed during the current study are not publicly available due to regulations set forth by the ethics boards. The dataset supporting the conclusions are included in the results section of this manuscript and the corresponding supplementary material. The comprehensive datasets are available from the corresponding author on reasonable request.

#### Competing interests

Dr. Fitzsimmons-Craft receives royalties from UpToDate, is on the Clinical Advisory Board for Beanbag Health, and is a consultant for Kooth. The other authors of this paper do not have any conflicts of interest to disclose.

#### Funding

This research was supported by a Feeding Hope Grant from the National Eating Disorders Association, as well as K08 MH120341 and K23 MH120347 from the National Institute of Mental Health. Participants in part were recruited through the National Eating Disorders Association's website.

#### Authors' contributions

SSV: Methodology, Software, Formal analysis, Investigation, Resources, Data Curation, Writing – Original draft preparation, Visualization, Supervision, Project Administration

AAL: Methodology, Formal analysis, Data curation, Writing – Original draft preparation, Visualization, Supervision

PC: Methodology, Formal analysis, Data curation, Writing – Reviewing & Editing, Visualization, Project administration

CGM: Formal analysis, Data curation, Writing – Reviewing & Editing

MF: Formal analysis, Data curation, Writing – Reviewing & Editing

DEW: Conceptualization, Writing – Reviewing & Editing,

CBT: Conceptualization, Writing – Reviewing & Editing,

EEFC: Conceptualization, Methodology, Formal analysis, Investigation, Resources, Writing – Original draft preparation, Visualization, Supervision, Project Administration, Funding Acquisition

ECA: Conceptualization, Methodology, Formal analysis, Investigation, Resources, Writing – Original draft preparation, Visualization, Supervision, Project Administration, Funding Acquisition

#### Acknowledgements

We sincerely thank all of the participants who made this trial possible.

#### Authors' information

Not Applicable.

#### Footnotes

Not Applicable.

### Figures

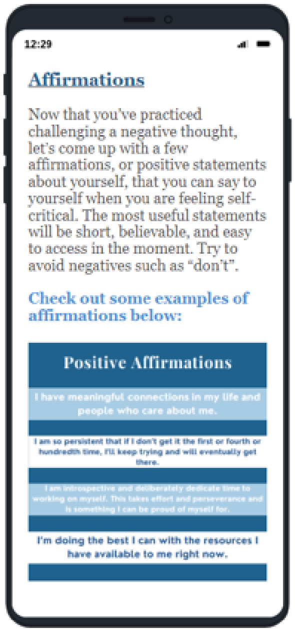

**Figure 1**

Cognitive-Behavioral Therapy-informed educational content.

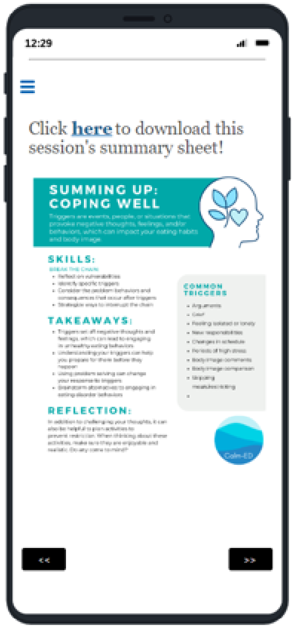

**Figure 2**

Downloadable PDF summary of session content.

### Supplementary Files

This is a list of supplementary files associated with this preprint. Click to download.

- VendlinskiInterviewSupplement.docx
- VendlinskiTableSupplement.docx
